# Supplementary material for: Unveiling unique clinical phenotypes of hip fracture patients and the temporal association with cardiovascular events
Source: Nat Commun. 2024 May 22;15:4353. doi: 10.1038/s41467-024-48713-3 (PMC11111763; doi:10.1038/s41467-024-48713-3)

Unveiling Unique Clinical Phenotypes of Hip Fracture Patients and the Temporal Association with Cardiovascular Events in Hong Kong and the United Kingdom: A Retrospective Study

Supplementary Materials

Table of contents:

Name	Title	Page number
Supplementary Table 1	Baseline characteristics of the HK CDARS and UK THIN study cohorts.	2
Supplementary Table 2	Summary of class membership probability.	3
Supplementary Table 3	Pearson's correlation coefficients showing cluster similarity between HK CDARS training and test sets.	4
Supplementary Table 4	The association between hip fracture subphenotypes and 180-day individual MACE outcomes.	5
Supplementary Table 5	The association between hip fracture subphenotypes and 180-day outcomes of interest, excluding patients with MACE within 30 days after index date.	6
Supplementary Table 6	The association between hip fracture subphenotypes and 180-day cardiac A&E hospitalisation outcomes in HK CDARS.	7
Supplementary Table 7	The association between hip fracture subphenotypes and 180-day MACE defined by myocardial infarction and stroke.	7
Supplementary Table 8	Stratified analysis on the association between hip fracture subphenotypes and 180-day outcomes of interest.	8
Supplementary Table 9	Results of competing risk regression analysis on the association between hip fracture subphenotypes and risk of individual MACE outcomes at multiple time points.<br>(a) HK CDARS<br>(b) UK THIN	9-10
Supplementary Table 10	Results of the SCCS analysis (individual MACE outcomes and sensitivity analysis).<br>(a) HK CDARS<br>(b) UK THIN	11-13
Supplementary Table 11	Comparison of the event rates of the hip fracture subphenotypes with the MI reference cohort in HK CDARS.	14
Supplementary Table 12	Covariates included in the latent class analysis.	15
Supplementary Table 13	ICD-9 codes for the MACE outcomes.	16
Supplementary Figure 1	Cohort selection flowcharts.<br>(a) HK CDARS<br>(b) UK THIN	17-18
Supplementary Figure 2	Model selection statistics selecting optimal number of clusters.<br>(a) HK CDARS training set<br>(b) HK CDARS test set<br>(c) UK THIN Cohort	19-24
Supplementary Figure 3	The prevalence of baseline diagnoses of hip fracture subphenotypes in the datasets.<br>(a) HK CDARS training set<br>(b) HK CDARS test set<br>(c) UK THIN Cohort	25-27
Supplementary Figure 4	Hazard ratios for MACE at multiple time points from competing risk regression.<br>(a) HK CDARS: Cluster 1 vs 3<br>(b) HK CDARS: Cluster 2 vs 3<br>(c) UK THIN Cohort: Cluster 1 vs 2	28-30
Supplementary Figure 5	Association between hip fracture and individual MACE outcomes through SCCS.<br>(a) HK CDARS<br>(b) UK THIN	31-32
Supplementary Figure 6	Incidence Rates for MACE by Cluster in the Hip Fracture Cohorts.<br>(a) HK CDARS<br>(b) UK THIN	33-34
Supplementary Figure 7	Schematic plot of the SCCS design.	35

Supplementary Table 1. Baseline characteristics of the HK CDARS and UK THIN study cohorts.

	Cohorts	
**Variables**	HK CDARS	UK THIN
N	78417	27948
Males, n (%)	24594 (31.4)	7184 (25.7)
Age, mean (SD)	83.43 (7.52)	82.48 (7.67)
**Diagnosis record within 5 years before index date, n (%)**		
Coronary heart disease	8708 (11.1)	3237 (11.6)
Congestive heart failure	7605 (9.7)	1502 (5.4)
Cerebrovascular diseases	8723 (11.1)	2847 (10.2)
Hypertensive diseases	29406 (37.5)	3655 (13.1)
Arrhythmia and conduction disorders	9362 (11.9)	3371 (12.1)
Arterial disease	2925 (3.7)	1427 (5.1)
Chronic obstructive pulmonary disease	7126 (9.1)	3050 (10.9)
Hyperlipidemia	6893 (8.8)	1082 (3.9)
Obesity	355 (0.5)	343 (1.2)
Diabetes	15238 (19.4)	1852 (6.6)
Thyroid disorders	1457 (1.9)	2645 (9.5)
Chronic renal disease	4258 (5.4)	5008 (17.9)
Liver diseases	509 (0.6)	99 (0.4)
Osteoporosis	3028 (3.9)	3217 (11.5)
Paget’s disease of bone	18 (0.0)	50 (0.2)
Major fractures other than hip fracture	6399 (8.2)	3004 (10.7)
Connective tissue disease	548 (0.7)	1037 (3.7)
Osteoarthritis	5720 (7.3)	4940 (17.7)
Depression	1344 (1.7)	1856 (6.6)
Dementia	5612 (7.2)	3088 (11.0)

Notes: % = Percentage; SD = Standard Deviation.

Supplementary Table 2. Summary of class membership probability.

Cohort	Group	1st Quartile	Median	3rd Quartile
HK CDARS training set	Cluster 1	0.73	0.85	0.93
	Cluster 2	0.76	0.94	0.99
	Cluster 3	0.87	0.98	0.99
HK CDARS test set	Cluster 1	0.73	0.82	0.93
	Cluster 2	0.72	0.92	0.99
	Cluster 3	0.85	0.98	0.98
UK THIN	Cluster 1	0.64	0.82	0.97
	Cluster 2	0.81	0.91	0.95

Class membership probabilities are computed for each subject through LCA. This probability represents the likelihood that a subject would be assigned to a certain class (computed using the subject's profile defined by the clustering variables). For example, within the context of a three-cluster LCA solution, each patient is associated with three class membership probabilities, corresponding to the likelihood of their belonging to each of the three latent classes. These probabilities quantify the confidence in class assignment, with higher values indicating greater certainty that a patient aligns with a particular class. Patients are assigned into the class for which they hold the highest membership probability.

Supplementary Table 3. Pearson's correlation coefficients showing cluster similarity between HK CDARS training and test sets.

	Test Set Cluster 1	Test Set Cluster 2	Test Set Cluster 3
Training Set Cluster 1	1.00	0.72	0.54
Training Set Cluster 2	0.66	1.00	0.57
Training Set Cluster 3	0.53	0.61	1.00

Notes: Values highlighted in grey showing high correlation between the corresponding clusters in training and test sets.

For each cluster identified, latent class analysis computed a set of conditional probabilities associated with the 22 clustering variables considered in this study. These conditional probabilities, also known as item-response probabilities, were estimated through the maximum likelihood estimation process in LCA. The item-response probabilities are the likelihood of observing a specific response given a latent class. For example, a conditional probability of 0.598 for baseline heart failure in cluster 2 in HK CDARS implies that there is a 59.8% chance that a patient classified to cluster 2 has baseline heart failure.

Each cluster could therefore be characterised by a list of 22 conditional probability values (one for each clustering variable), that could be leveraged for cluster comparison. For example, to assess the similarity between Cluster 1 in the training set and Cluster 1 in the test set, the Pearson's correlation coefficient was computed using their respective lists of 22 conditional probabilities. This process was repeated for each pair of clusters between the training and test sets.

Higher correlation coefficients towards 1 indicate higher degree of similarity between the two clusters being compared (a similar profile regarding the presence of the clustering variables). The results from Supplementary Table 3 showed consistency of cluster characteristics between the corresponding clusters across the training and test sets.

Supplementary Table 4. The association between hip fracture subphenotypes and 180-day individual MACE outcomes.

	HK CDARS				UK THIN	
	Cluster 1 vs 3 (ref)		Cluster 2 vs 3 (ref)		Cluster 1 vs 2 (ref)	
**Clinical Outcomes[a]**	Hazard Ratio (95% CI)	p-value	Hazard Ratio (95% CI)	p-value	Hazard Ratio (95% CI)	p-value
Heart Failure Hospitalisation	1.48 (1.26 – 1.75)	<0.001	9.66 (8.58 – 10.88)	<0.001	NA	NA
Myocardial Infarction	2.00 (1.73 – 2.31)	<0.001	3.70 (3.22 – 4.26)	<0.001	2.14 (1.61 to 2.85)	<0.001
Stroke	2.17 (1.97 – 2.39)	<0.001	1.79 (1.58 – 2.02)	<0.001	1.67 (1.30 to 2.14)	<0.001

Notes: CI = Confidence Interval.

[a]Hazard Ratios, associated 95% CIs and two-sided p-values are derived from competing risk regression, with adjustment of age and sex.

Supplementary Table 5. The association between hip fracture subphenotypes and 180-day outcomes of interest, excluding patients with MACE within 30 days after index date.

	HK CDARS[a]				UK THIN[b]	
	Cluster 1 vs 3 (ref)		Cluster 2 vs 3 (ref)		Cluster 1 vs 2 (ref)	
**Clinical Outcomes**	Hazard Ratio (95% CI)	p-value	Hazard Ratio (95% CI)	p-value	Hazard Ratio (95% CI)	p-value
All-cause Mortality[c]	1.32 (1.25 – 1.40)	<0.001	2.14 (2.02 – 2.26)	<0.001	1.38 (1.28 to 1.49)	<0.001
MACE[d]	1.85 (1.69 – 2.02)	<0.001	4.29 (3.95 – 4.67)	<0.001	1.86 (1.47 to 2.36)	<0.001
Heart Failure Hospitalisation[d]	1.43 (1.19 – 1.73)	<0.001	9.88 (8.64 – 11.29)	<0.001	NA	NA
Myocardial Infarction[d]	1.87 (1.56 – 2.24)	<0.001	3.04 (2.54 – 3.63)	<0.001	2.31 (1.53 to 3.49)	<0.001
Stroke[d]	2.07 (1.84 – 2.33)	<0.001	1.84 (1.58 – 2.14)	<0.001	1.72 (1.29 to 2.29)	<0.001
**Hospital Outcomes[e]**	Incidence Rate Ratio (95% CI)	p-value	Incidence Rate Ratio (95% CI)	p-value	Incidence Rate Ratio (95% CI)	p-value
Number of hospital visits	1.49 (1.47 – 1.51)	<0.001	2.02 (1.98 – 2.06)	<0.001	NA	NA
Number of A&E visits	1.47 (1.44 – 1.50)	<0.001	2.01 (1.96 – 2.06)	<0.001	NA	NA
Total length of hospital stays in days	1.31 (1.30 – 1.31)	<0.001	1.72 (1.71 – 1.73)	<0.001	NA	NA

Notes: MACE = Major Adverse Cardiovascular Events; A&E = Accident and Emergency; CI = Confidence Interval.
[a]n = 77,056 independent patients in the HK CDARS after removal of patients with MACE within 30 days after index date.
[b]n = 27,754 independent patients in the UK THIN after removal of patients with MACE within 30 days after index date.
[c]Hazard Ratios, associated 95% CIs and two-sided p-values are derived from Cox proportional regression, with adjustment of age and sex.
[d]Hazard Ratios, associated 95% CIs and two-sided p-values are derived from competing risk regression, with adjustment of age and sex.
[e]Incidence Rate Ratios, associated 95% CIs and two-sided p-values are derived from Poisson regression, with adjustment of age and sex.

Supplementary Table 6. The association between hip fracture subphenotypes and 180-day cardiac A&E hospitalisation outcomes in HK CDARS.

	Cluster 1 vs 3 (ref)		Cluster 2 vs 3 (ref)	
**Clinical Outcomes[a]**	Hazard Ratio (95% CI)	p-value	Hazard Ratio (95% CI)	p-value
MACE Hospitalisation	1.68 (1.52 – 1.86)	<0.001	5.36 (4.92 – 5.84)	<0.001
Heart Failure Hospitalisation	1.48 (1.26 – 1.75)	<0.001	9.66 (8.58 – 10.88)	<0.001
Myocardial Infarction Hospitalisation	2.12 (1.69 – 2.65)	<0.001	3.95 (3.17 – 4.91)	<0.001
Stroke Hospitalisation	1.70 (1.46 – 1.97)	<0.001	1.87 (1.57 – 2.22)	<0.001

Notes: MACE = Major Adverse Cardiovascular Events; CI = Confidence Interval.
[a]Hazard Ratios, associated 95% CIs and two-sided p-values are derived from competing risk regression, with adjustment of age and sex.

Supplementary Table 7. The association between hip fracture subphenotypes and 180-day MACE defined by myocardial infarction and stroke.

	HK CDARS				UK THIN	
	Cluster 1 vs 3 (ref)		Cluster 2 vs 3 (ref)		Cluster 1 vs 2 (ref)	
**Clinical Outcome[a]**	Hazard Ratio (95% CI)	p-value	Hazard Ratio (95% CI)	p-value	Hazard Ratio (95% CI)	p-value
MACE (defined by MI and Stroke)	2.09 (1.93 – 2.27)	<0.001	2.38 (2.17 – 2.61)	<0.001	1.84 (1.53 – 2.23)	<0.001

Notes: MACE = Major Adverse Cardiovascular Events; MI = Myocardial Infarction; CI = Confidence Interval.
[a]Hazard Ratios, associated 95% CIs and two-sided p-values are derived from competing risk regression, with adjustment of age and sex.

Supplementary Table 8. Stratified analysis on the association between hip fracture subphenotypes and 180-day outcomes of interest.

		HK CDARS						UK THIN		
		Cluster 1 vs 3 (ref)			Cluster 2 vs 3 (ref)			Cluster 1 vs 2 (ref)		
**Clinical Outcomes**	**Group**	HR (95% CI)	p-value	$P_{interaction}$	HR (95% CI)	p-value	$P_{interaction}$	HR (95% CI)	p-value	$P_{interaction}$
All-cause Mortality[a]	Female[c]	1.45 (1.35 – 1.57)	<0.001	0.0116	2.51 (2.34 – 2.70)	<0.001	<0.001	1.47 (1.33 to 1.62)	<0.001	0.10
	Male[c]	1.25 (1.15 – 1.35)	<0.001		1.93 (1.78 – 2.08)	<0.001		1.28 (1.14 to 1.44)	<0.001	
	Older[d]	1.20 (1.11 – 1.30)	<0.001	<0.001	2.00 (1.87 – 2.14)	<0.001	<0.001	1.29 (1.17 to 1.42)	<0.001	<0.001
	Younger[d]	1.49 (1.38 – 1.61)	<0.001		2.90 (2.66 – 3.16)	<0.001		1.63 (1.45 to 1.83)	<0.001	
	Partial Hip Replacement[e]	1.48 (1.27 – 1.72)	<0.001	0.12	2.33 (1.99 – 2.72)	<0.001	0.0376	NA	NA	NA
	Internal fixation[e]	1.27 (1.14 – 1.41)	<0.001		1.99 (1.79 – 2.21)	<0.001		NA	NA	
MACE[b]	Female[c]	1.89 (1.73 – 2.08)	<0.001	0.14	4.37 (4.01 – 4.75)	<0.001	<0.001	2.18 (1.74 to 2.73)	<0.001	0.018
	Male[c]	2.10 (1.85 – 2.37)	<0.001		3.49 (3.08 – 3.95)	<0.001		1.35 (0.98 to 1.86)	0.07	
	Older[d]	1.65 (1.48 – 1.85)	<0.001	<0.001	3.51 (3.21 – 3.84)	<0.001	<0.001	1.87 (1.43 to 2.44)	<0.001	0.85
	Younger[d]	2.29 (2.07 – 2.54)	<0.001		5.25 (4.72 – 5.84)	<0.001		1.85 (1.41 to 2.42)	<0.001	
	Partial Hip Replacement[e]	2.04 (1.67 – 2.48)	<0.001	0.41	4.34 (3.58 – 5.27)	<0.001	0.15	NA	NA	NA
	Internal fixation[e]	1.86 (1.62 – 2.13)	<0.001		3.73 (3.26 – 4.27)	<0.001		NA	NA	

Notes: HR = Hazard Ratio; MACE = Major Adverse Cardiovascular Events; CI = Confidence Interval.
[a]Hazard Ratios, associated 95% CIs and two-sided p-values are derived from Cox proportional regression.
[b]Hazard Ratios, associated 95% CIs and two-sided p-values are derived from competing risk regression.
[c]Analysis adjusted for age.
[d]Older age group defined by 85 years or above, whereas younger age group was defined by below 85 years. Analysis adjusted for sex.
[e]Internal fixation (n = 24,526 independent patients) and partial hip replacement (n = 14,042 independent patients) were the two most common procedures recorded 7 days before or after the hip fracture event date. Analysis adjusted for age and sex.

Supplementary Table 9. Results of competing risk regression analysis on the association between hip fracture subphenotypes and risk of individual MACE outcomes at multiple time points.
(a) HK CDARS

	Comparison	90 days	180 days	270 days	1 year
Heart Failure Hospitalisation	Cluster 1 vs 3 (ref)	1.40<br>(1.12 – 1.73)<br>p = 0.0026	1.48<br>(1.26 – 1.75)<br>p < 0.001	1.44<br>(1.25 – 1.66)<br>p < 0.001	1.47<br>(1.30 – 1.67)<br>p < 0.001
	Cluster 2 vs 3 (ref)	9.98<br>(8.56 – 11.62)<br>p < 0.001	9.66<br>(8.58 – 10.88)<br>p < 0.001	9.23<br>(8.33 – 10.24)<br>p < 0.001	8.78<br>(7.99 – 9.64)<br>p < 0.001
Myocardial Infarction	Cluster 1 vs 3 (ref)	2.04<br>(1.71 – 2.44)<br>p < 0.001	2.00<br>(1.73 – 2.31)<br>p < 0.001	2.00<br>(1.75 – 2.28)<br>p < 0.001	1.97<br>(1.75 – 2.22)<br>p < 0.001
	Cluster 2 vs 3 (ref)	3.85<br>(3.25 – 4.56)<br>p < 0.001	3.70<br>(3.22 – 4.26)<br>p < 0.001	3.63<br>(3.20 – 4.12)<br>p < 0.001	3.63<br>(3.24 – 4.08)<br>p < 0.001
Stroke	Cluster 1 vs 3 (ref)	2.37<br>(2.12 – 2.65)<br>p < 0.001	2.17<br>(1.97 – 2.39)<br>p < 0.001	2.06<br>(1.89 – 2.24)<br>p < 0.001	1.97<br>(1.82 – 2.13)<br>p < 0.001
	Cluster 2 vs 3 (ref)	1.81<br>(1.56 – 2.09)<br>p < 0.001	1.79<br>(1.58 – 2.02)<br>p < 0.001	1.71<br>(1.53 – 1.91)<br>p < 0.001	1.78<br>(1.62 – 1.96)<br>p < 0.001

Note: Data are presented as Hazard Ratio (95% Confidence Interval) two-sided p-value derived from competing risk regression. All analyses are adjusted for age and sex.

(b) UK THIN

	Comparison	90 days	180 days	270 days	1 year
Myocardial Infarction	Cluster 1 vs 2 (ref)	2.00<br>(1.43 – 2.79)<br>p < 0.001	2.14<br>(1.61 – 2.85)<br>p < 0.001	2.12<br>(1.64 – 2.74)<br>p < 0.001	2.11<br>(1.66 to 2.68)<br>p < 0.001
Stroke	Cluster 1 vs 2 (ref)	1.80<br>(1.31 – 2.48)<br>p < 0.001	1.67<br>(1.30 – 2.14)<br>p < 0.001	1.52<br>(1.22 – 1.88)<br>p < 0.001	1.45<br>(1.19 to 1.76)<br>p < 0.001

Note: Data are presented as Hazard Ratio (95% Confidence Interval) two-sided p-value derived from competing risk regression. All analyses are adjusted for age and sex.

Supplementary Table 10. Results of the SCCS analysis (individual MACE outcomes and sensitivity analysis).
(a) HK CDARS

	Cluster 1		Cluster 2		Cluster 3		Whole	
Hip fracture exposure window, d	No. of events	IRR[a] (95% CI)	No. of events	IRR[a] (95% CI)	No. of events	IRR[a] (95% CI)	No. of events	IRR[a] (95% CI)
**Heart failure hospitalisation**	332		1249		779		2360	
1-60 d	31	1.41 (0.97, 2.06)	71	1.65 (1.27, 2.13)	83	1.70 (1.35, 2.16)	185	1.54 (1.32, 1.81)
61-120 d	29	1.30 (0.88, 1.92)	66	1.58 (1.22, 2.06)	67	1.36 (1.05, 1.76)	162	1.36 (1.15, 1.60)
121-180 d	26	1.14 (0.76, 1.71)	46	1.13 (0.83, 1.54)	58	1.16 (0.89, 1.53)	130	1.09 (0.91, 1.31)
Baseline period[b]	246	1 (Reference)	1066	1 (Reference)	571	1 (Reference)	1883	1 (Reference)
**Myocardial infarction**	270		314		449		1033	
1-60 d	25	1.77 (1.15, 2.72)	21	1.60 (0.99, 2.57)	52	1.94 (1.43, 2.62)	98	1.75 (1.41, 2.18)
61-120 d	18	1.26 (0.77, 2.06)	11	0.86 (0.47, 1.59)	42	1.54 (1.11, 2.13)	71	1.26 (0.99, 1.62)
121-180 d	11	0.76 (0.41, 1.40)	11	0.88 (0.48, 1.64)	32	1.15 (0.80, 1.67)	54	0.95 (0.72, 1.26)
Baseline period[b]	216	1 (Reference)	271	1 (Reference)	323	1 (Reference)	810	1 (Reference)
**Stroke**	1550		465		1597		3612	
1-60 d	98	2.07 (1.65, 2.59)	39	1.84 (1.30, 2.61)	182	2.08 (1.77, 2.45)	319	1.93 (1.71, 2.18)
61-120 d	39	0.85 (0.61, 1.18)	25	1.20 (0.79, 1.82)	112	1.27 (1.04, 1.54)	176	1.07 (0.92, 1.25)
121-180 d	35	0.79 (0.56, 1.12)	24	1.16 (0.76, 1.78)	85	0.95 (0.76, 1.18)	144	0.88 (0.74, 1.05)
Baseline period[b]	1378	1 (Reference)	377	1 (Reference)	1218	1 (Reference)	2973	1 (Reference)
**Applying shorter time intervals for risk periods: MACE**	2005		1695		2666		6366	
1-30 d	79	1.98 (1.57, 2.49)	58	2.01 (1.53, 2.66)	182	2.39 (2.05, 2.80)	319	2.17 (1.93, 2.44)
31-60 d	67	1.70 (1.32, 2.18)	56	1.97 (1.49, 2.60)	126	1.64 (1.37, 1.97)	249	1.69 (1.49, 1.93)
61-90 d	38	0.97 (0.70, 1.35)	48	1.70 (1.27, 2.29)	123	1.59 (1.32, 1.91)	209	1.42 (1.24, 1.64)
91-120 d	32	0.83 (0.58, 1.18)	38	1.36 (0.98, 1.89)	88	1.13 (0.91, 1.40)	158	1.08 (0.92, 1.26)
121-150 d	33	0.86 (0.61, 1.22)	33	1.20 (0.84, 1.71)	80	1.02 (0.82, 1.28)	146	0.99 (0.84, 1.18)
151-180 d	29	0.76 (0.53, 1.10)	30	1.10 (0.76, 1.60)	81	1.03 (0.82, 1.29)	140	0.95 (0.81, 1.13)
Baseline period[b]	1727	1 (Reference)	1432	1 (Reference)	1986	1 (Reference)	5145	1 (Reference)
**Considering only post-hip fracture period as the baseline: MACE**	1127		1073		2325		4525	
1-60 d	190	2.25 (1.9, 2.65)	181	2.48 (2.08, 2.97)	321	1.85 (1.63, 2.09)	692	2.43 (2.23, 2.64)
61-120 d	96	1.14 (0.92, 1.42)	139	1.92 (1.59, 2.32)	218	1.25 (1.08, 1.45)	453	1.56 (1.41, 1.73)

121-180 d	75	0.89 (0.7, 1.13)	106	1.47 (1.2, 1.81)	164	0.94 (0.8, 1.11)	345	1.17 (1.04, 1.31)
Baseline period[c]	766	1 (Reference)	647	1 (Reference)	1622	1 (Reference)	3035	1 (Reference)

Notes: MACE = Major Adverse Cardiovascular Events; d = day; IRR = Incidence Rate Ratios; CI = Confidence Interval.

[a]IRR, incidence rate ratio adjusted for age by quintiles.

[b]Baseline period indicates 366 days before hip fracture plus 181 to 732 days after hip fracture.

[c]Baseline period indicates 181 to 732 days after hip fracture.

(b) UK THIN

	Cluster 1		Cluster 2		Whole	
Hip fracture exposure window, d	No. of events	IRR[a] (95% CI)	No. of events	IRR[a] (95% CI)	No. of events	IRR[a] (95% CI)
**Myocardial infarction**	129		196		325	
1-60 d	24	6.76 (3.86, 11.84)	43	4.17 (2.90, 6.00)	67	4.56 (3.39, 6.13)
61-120 d	6	1.74 (0.71, 4.26)	12	1.14 (0.62, 2.09)	18	1.23 (0.75, 2.01)
121-180 d	4	1.16 (0.40, 3.36)	14	1.31 (0.75, 2.31)	18	1.23 (0.75, 2.02)
Baseline period[b]	95	1 (Reference)	127	1 (Reference)	222	1 (Reference)
**Stroke**	268		661		929	
1-60 d	15	1.89 (1.06, 3.36)	59	1.74 (1.32, 2.30)	74	1.74 (1.35, 2.23)
61-120 d	11	1.42 (0.75, 2.70)	30	0.88 (0.60, 1.28)	41	0.97 (0.70, 1.33)
121-180 d	12	1.62 (0.87, 3.03)	34	0.99 (0.69, 1.41)	46	1.08 (0.80, 1.47)
Baseline period[b]	230	1 (Reference)	538	1 (Reference)	768	1 (Reference)
**Applying shorter time intervals for risk periods: MACE**	382		844		1226	
1-30 d	27	4.72 (3.05, 7.29)	63	2.87 (2.20, 3.75)	90	3.20 (2.55, 4.01)
31-60 d	11	1.96 (1.04, 3.67)	36	1.64 (1.16, 2.30)	47	1.67 (1.24, 2.26)
61-90 d	10	1.80 (0.93, 3.47)	23	1.04 (0.68, 1.59)	33	1.18 (0.83, 1.67)
91-120 d	4	0.73 (0.27, 1.98)	19	0.86 (0.54, 1.36)	23	0.82 (0.54, 1.25)
121-150 d	8	1.48 (0.72, 3.04)	27	1.22 (0.83, 1.80)	35	1.25 (0.89, 1.76)
151-180 d	8	1.51 (0.73, 3.13)	20	0.90 (0.57, 1.41)	28	1.00 (0.69, 1.47)
Baseline period[b]	314	1 (Reference)	656	1 (Reference)	970	1 (Reference)
**Considering only post-hip fracture period as the baseline: MACE**	181		637		818	
1-60 d	43	4.60 (3.15, 6.73)	105	2.52 (2.02, 3.13)	148	2.80 (2.32, 3.38)
61-120 d	16	1.68 (0.98, 2.88)	45	1.06 (0.77, 1.44)	61	1.14 (0.87, 1.48)
121-180 d	19	1.95 (1.18, 3.21)	52	1.20 (0.90, 1.60)	71	1.30 (1.01, 1.67)
Baseline period[c]	103	1 (Reference)	435	1 (Reference)	538	1 (Reference)

Notes: MACE = Major Adverse Cardiovascular Events; d = day; IRR = Incidence Rate Ratios; CI = Confidence Interval.
[a]IRR, incidence rate ratio adjusted for age by quintiles.
[b]Baseline period indicates 366 days before hip fracture plus 181 to 732 days after hip fracture.
[c]Baseline period indicates 181 to 732 days after hip fracture.

Supplementary Table 11. Comparison of the event rates of the hip fracture subphenotypes with the MI reference cohort in HK CDARS.

	Cluster 1	Cluster 2	Cluster 3	Total	MI Reference Cohort
N	16,762	9,860	51,795	78,417	36,933
Baseline MI	3.42%	15.04%	0.85%	3.18%	100%
**MACE***					
60-days	3.48%	4.11%	1.56%	2.29%	18.89%
1-year	8.33%	10.72%	4.42%	6.05%	24.27%
**All-cause mortality**					
60-days	4.53%	9.59%	3.78%	4.67%	11.77%
1-year	17.25%	31.35%	13.57%	16.59%	27.99%

Notes: MACE = Major Adverse Cardiovascular; MI = Myocardial infarction.
*MACE defined by MI and stroke (as heart failure hospitalisation data were not available in the MI Reference Cohort).

Supplementary Table 12. Covariates included in the latent class analysis.

**Demographics**	
Sex	
Age	
**Diagnoses**	**ICD9 Codes**
Cardiovascular diseases	
Coronary heart disease	410, 411, 412, 413, 414, 429.2, 429.71, 429.79
Congestive heart failure	398.91, 402.01, 402.11, 402.91, 404.01, 404.03, 404.11, 404.13, 404.91, 404.93, 428
Cerebrovascular diseases	430-437
Hypertensive diseases	401-405
Arrhythmia and conduction disorders	426-427
Arterial disease	433.00, 433.10, 433.20, 433.30, 433.80, 433.90, 440-445, 447
Respiratory related diseases	
Chronic obstructive pulmonary disease	490-496, 500-505, 506.4
Endocrine and metabolic disorders	
Overweight and obesity	278.0
Hyperlipidaemia	272.0-272.2, 272.4
Diabetes	250
Thyroid disorders	242-244
Renal diseases	
Chronic renal disease	403, 404, 582, 585, 590.0
Liver diseases	
Esophageal varices, chronic liver disease, hepatic failure, cirrhosis	456.0, 456.1, 456.2, 571.2, 571.4, 571.5, 571.6, 572.2, 572.3, 572.4, 572.8
Bone related diseases	
Osteoporosis	733.0
Paget's disease of bone	731.0
Major fractures other than hip fracture	805, 812, 813, 814
Osteoarthritis	715, 721
Other diseases	
Dementia	290
Depression	296,2, 296.3
Connective tissue disease	710.0, 710.1, 710.4, 714.0, 714.1, 714.2, 714.81, 725

Supplementary Table 13. ICD-9 codes for the MACE outcomes.

Outcomes	ICD-9 Codes
Stroke	430, 431, 432, 433.01, 433.11, 433.21, 433.31, 433.81, 433.91, 434, 436, 437.0, 437.1
Myocardial Infarction	410
Heart failure	398.91, 402.01, 402.11, 402.91, 404.01, 404.03, 404.11, 404.13, 404.91, 404.93, 428

Supplementary Figure 1. Cohort selection flowcharts.
(a) HK CDARS

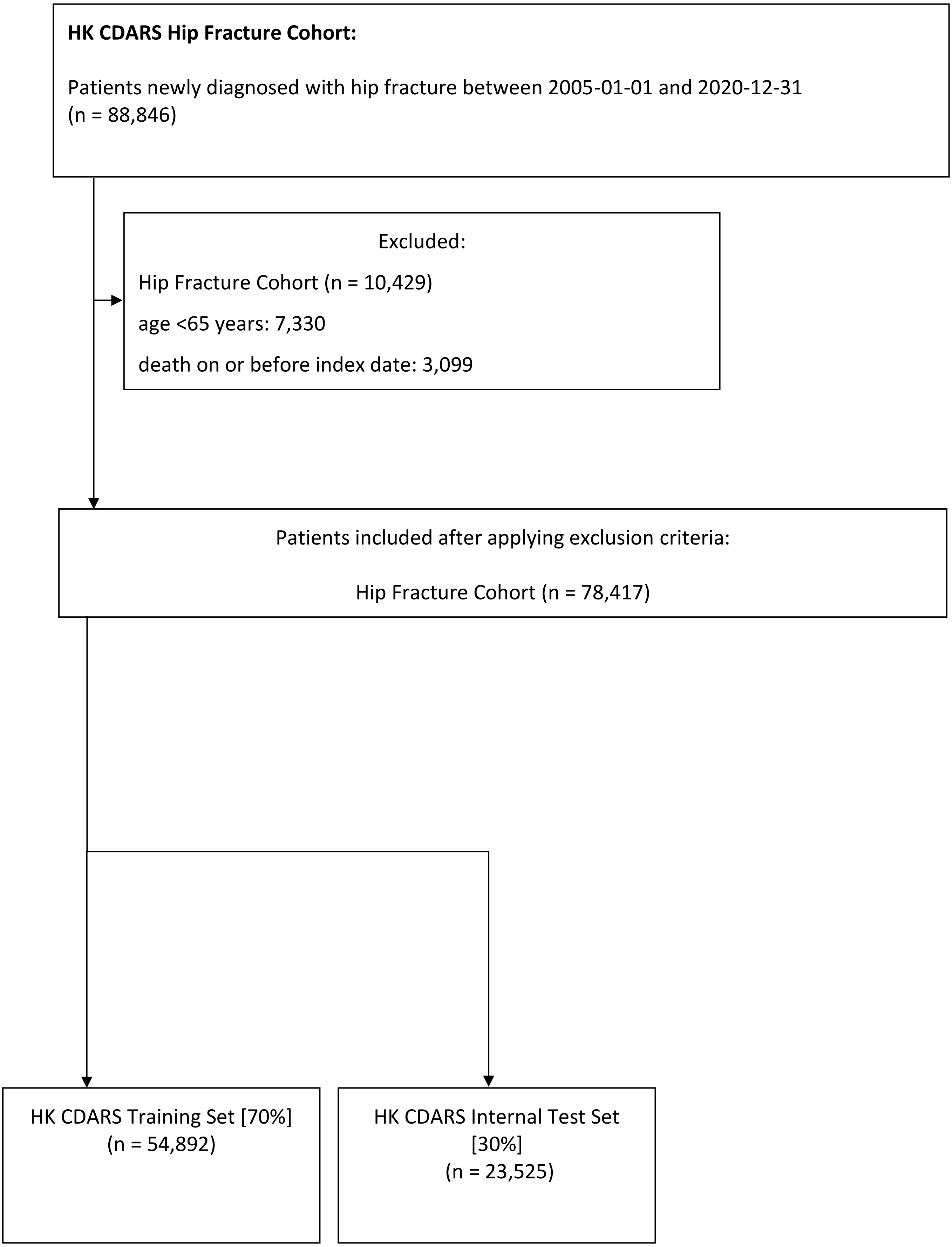

(b) UK THIN

UK THIN Hip Fracture Cohort:

Patients newly diagnosed with hip fracture between 2005-01-01 and 2018-12-31
(n = 32,158)

Excluded:

Hip Fracture Cohort (n = 4,210)

age <65 years: 4,072

death on or before index date: 138

Patients included after applying exclusion criteria:

Hip Fracture Cohort (n = 27,948)

Supplementary Figure 2. Model selection statistics selecting optimal number of clusters.
(a) HK CDARS training set

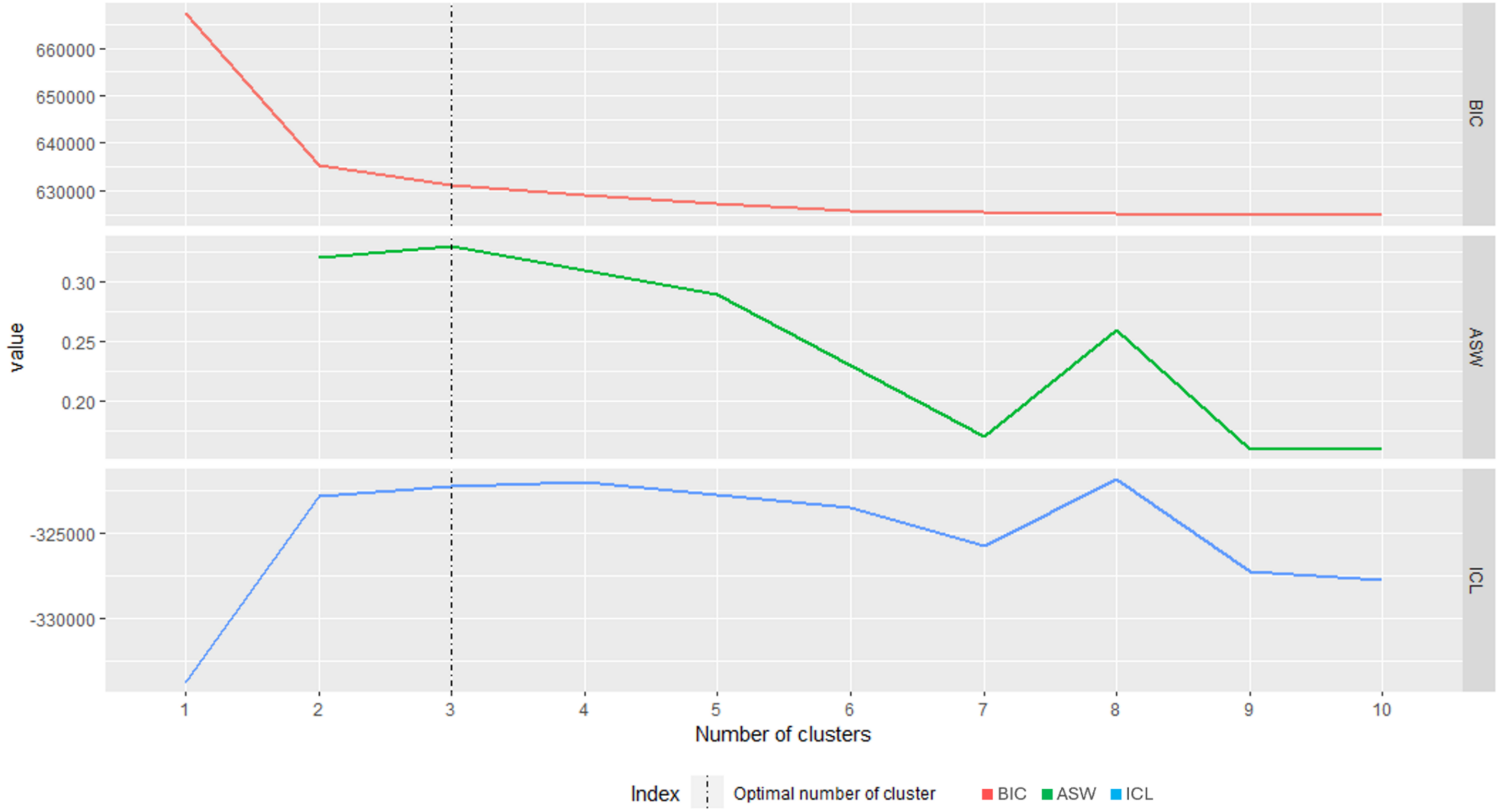

Number of Cluster	1	2	3	4	5	6	7	8	9	10
BIC	667364.74	635478.38	631096.74	629057.52	627369.77	625929.08	625341.05	625048.33	624872.25	624759.03
ASW	NA	0.32	0.33	0.31	0.29	0.23	0.17	0.26	0.16	0.16
ICL	-333687.34	-322806.71	-322271.16	-322032.98	-322781.29	-323527.67	-325732.38	-321848.15	-327226.58	-327768.00

n = 54,892 independent patients in HK CDARS training set.

HK CDARS training set: 3-clusters solution

Rationale: From the BIC plot, a distinct inflection point could be observed at approximately 2 to 3 clusters. representing a marked improvement in model fit. Although BIC showed a marginal decrease with additional clusters beyond the point of three clusters, the gain in model fit did not outweigh the increased complexity. Furthermore, the absolute value of ASW was the highest at 3 clusters, and ICL was also among the highest levels at 3 clusters. While the ICL achieved its absolute peak at 8 clusters, a solution with fewer clusters is preferable for clinical applicability and ease of interpretation. Therefore, a 3-clusters solution was selected as the optimal solution, striking a balance among BIC, ASW, and ICL, while maintaining clinical interpretability.

(b) HK CDARS test set

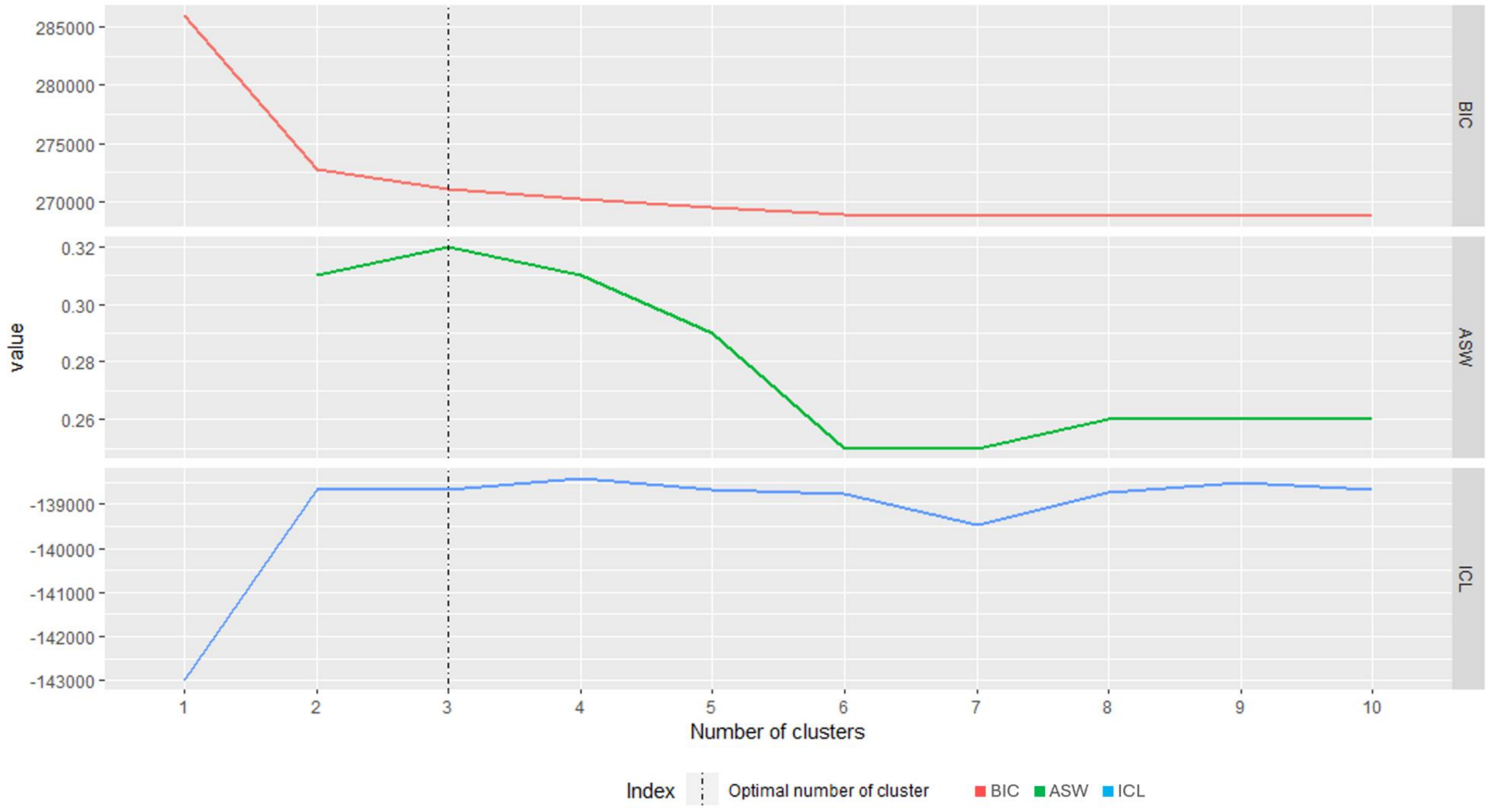

Number of Cluster	1	2	3	4	5	6	7	8	9	10
BIC	285948.27	272832.95	271075.09	270226.12	269517.88	268962.26	268819.73	268762.85	268790.42	268873.53
ASW	NA	0.31	0.32	0.31	0.29	0.25	0.25	0.26	0.26	0.26
ICL	-142979.11	-138674.61	-138657.79	-138413.92	-138664.04	-138758.25	-139454.37	-138718.76	-138522.07	-138652.54

n = 23,525 independent patients in HK CDARS test set.

HK CDARS test set: 3-clusters solution

Rationale: The trajectories of the three model performance metrics for HK CDARS test set resembled that of the training set. Similarly, for BIC, the marked inflection point was observed at approximately 2 to 3 clusters, with no substantial improvement in model fit beyond this range. The absolute value of ASW was again the highest at 3 clusters, and the ICL at 3 clusters was also among the highest levels. Therefore, 3-clusters was again an appropriate point of agreement across the three metrics and was selected as the final solution.

(c) UK THIN Cohort

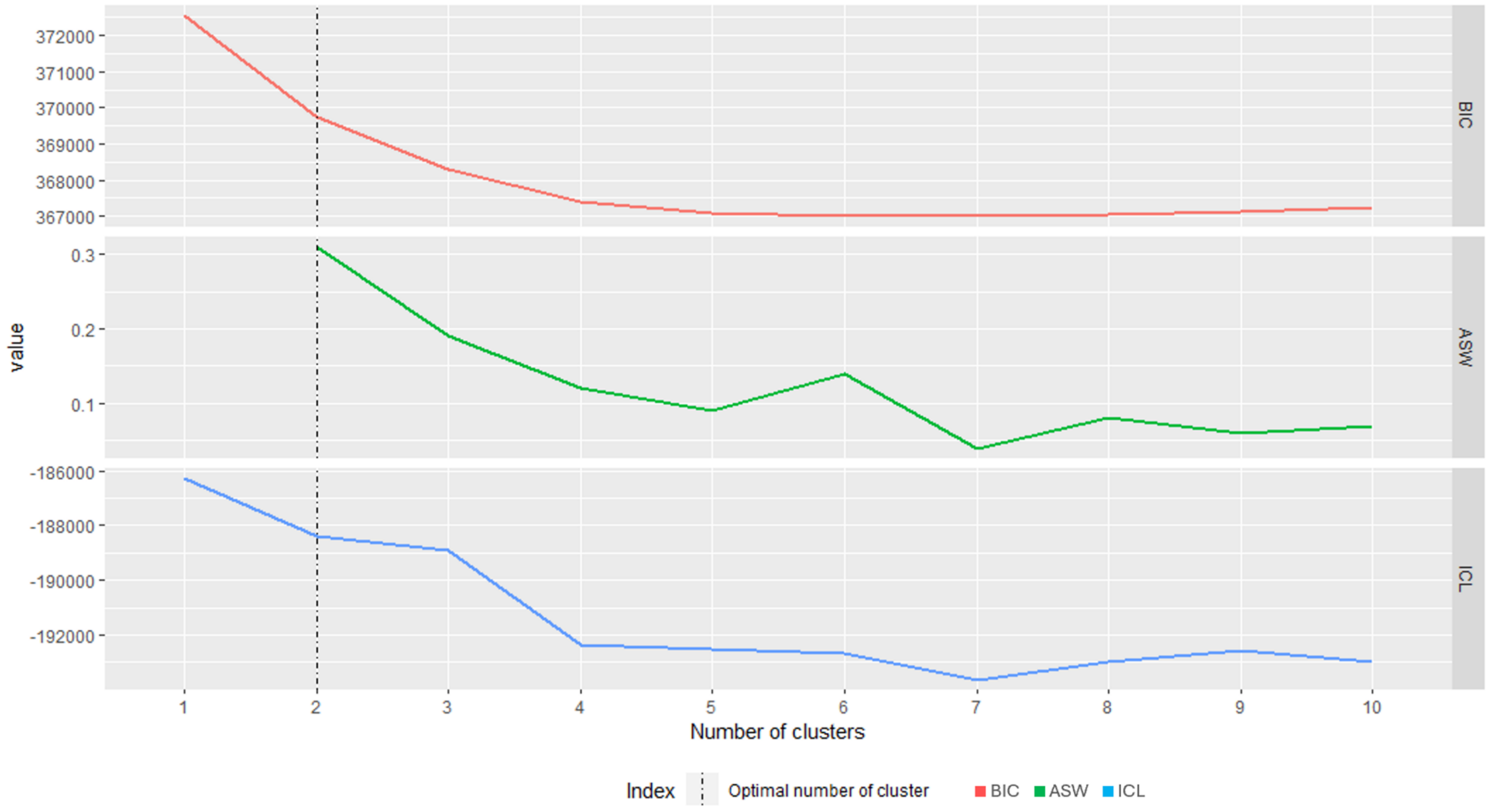

Number of Cluster	1	2	3	4	5	6	7	8	9	10
BIC	372557.98	369752.12	368310.79	367388.13	367074.43	366986.40	367017.36	367051.88	367110.76	367233.16
ASW	NA	0.31	0.19	0.12	0.09	0.14	0.04	0.08	0.06	0.07
ICL	-186283.96	-188404.57	-188918.03	-192356.07	-192526.08	-192690.11	-193624.19	-192979.51	-192572.36	-192999.71

n = 27,948 independent patients in UK THIN.

UK THIN Cohort: 2 clusters solution

Rationale: The BIC plot for the UK THIN cohort identified a marked inflection zone between 2 to 4 clusters. The absolute ASW value was the highest at 2 clusters, while the ICL value peaked at a single cluster, followed by a steady decline in ICL values with additional clusters. The downward trends in both ASW and ICL beyond two clusters suggested that a smaller number of clusters may better capture the data structure. Integrating all the three metrics led to our conclusion of evaluating a 2-clusters solution for the UK THIN cohort.

Supplementary Figure 3. The prevalence of baseline diagnoses of hip fracture subphenotypes in the datasets.
(a) HK CDARS training set

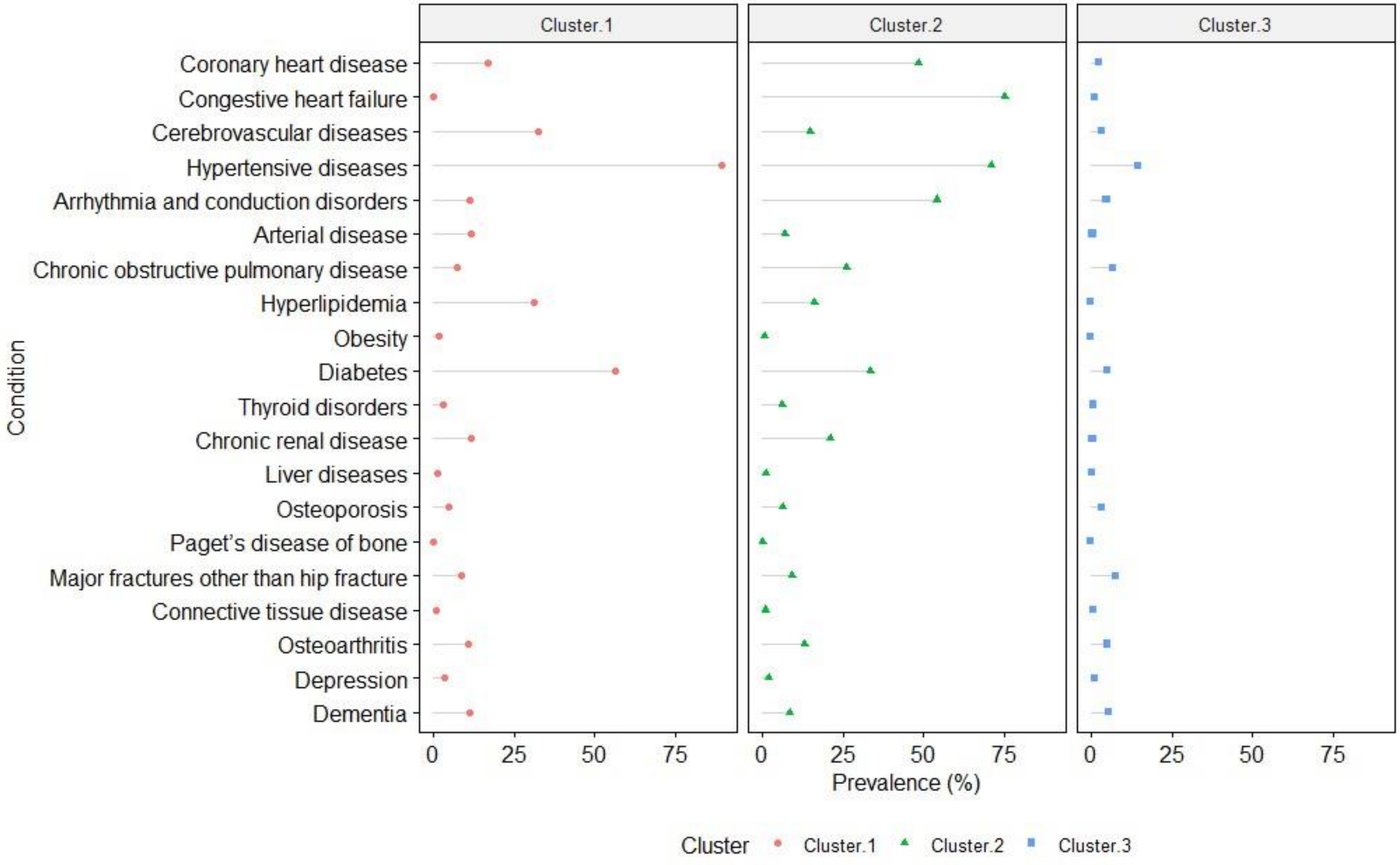

The Prevalence are indicated by the symbols (circle for Cluster 1; triangle for Cluster 2; Square for Cluster 3). n = 54,892 independent patients in HK CDARS training set (n = 11,886 in Cluster 1; n = 6,588 in Cluster 2; n = 36,418 in Cluster 3).

(b) HK CDARS test set

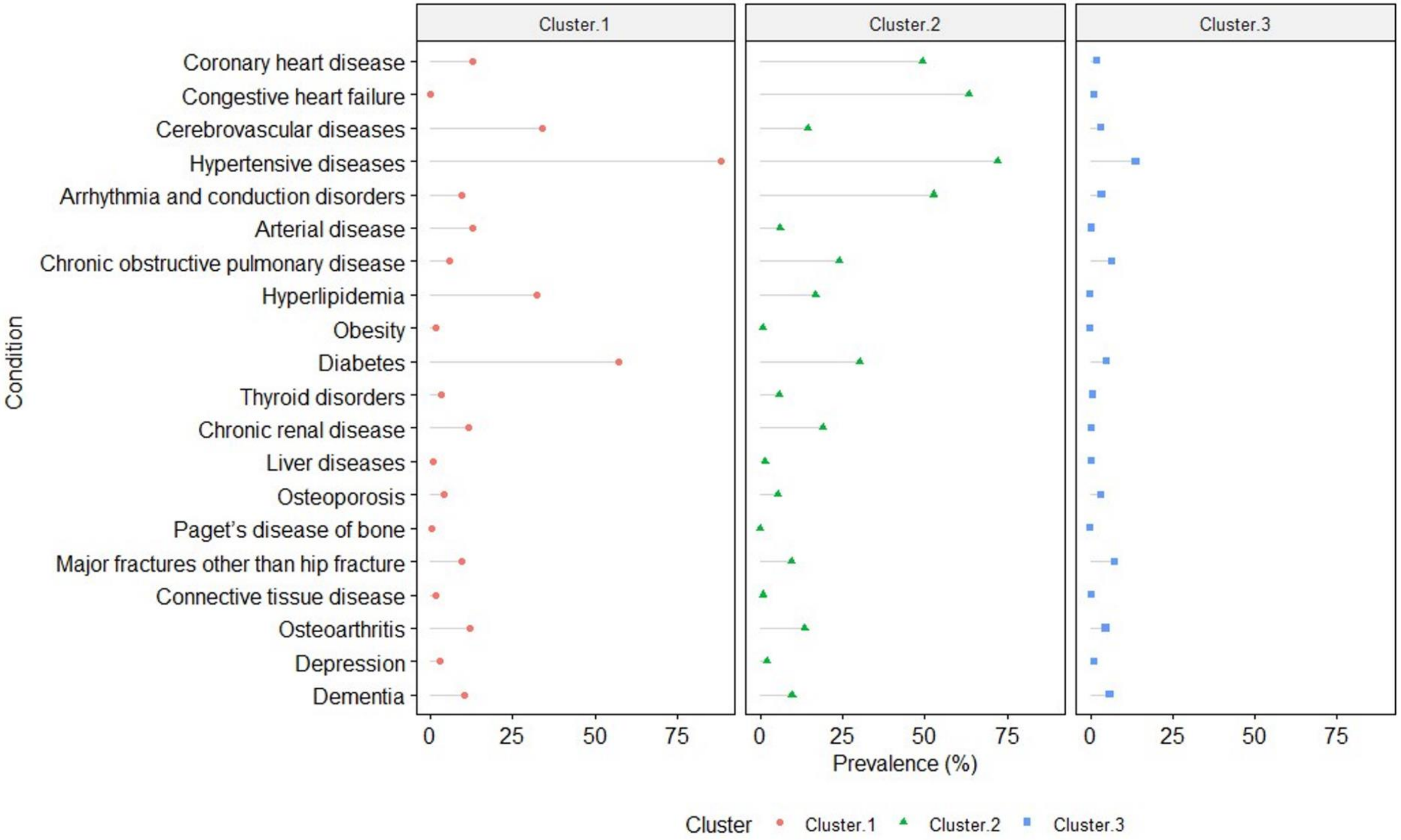

The Prevalence are indicated by the symbols (circle for Cluster 1; triangle for Cluster 2; Square for Cluster 3). n = 23,525 independent patients in HK CDARS test set (n = 4,876 in Cluster 1; n = 3,272 in Cluster 2; n = 15,377 in Cluster 3).

(c) UK THIN Cohort

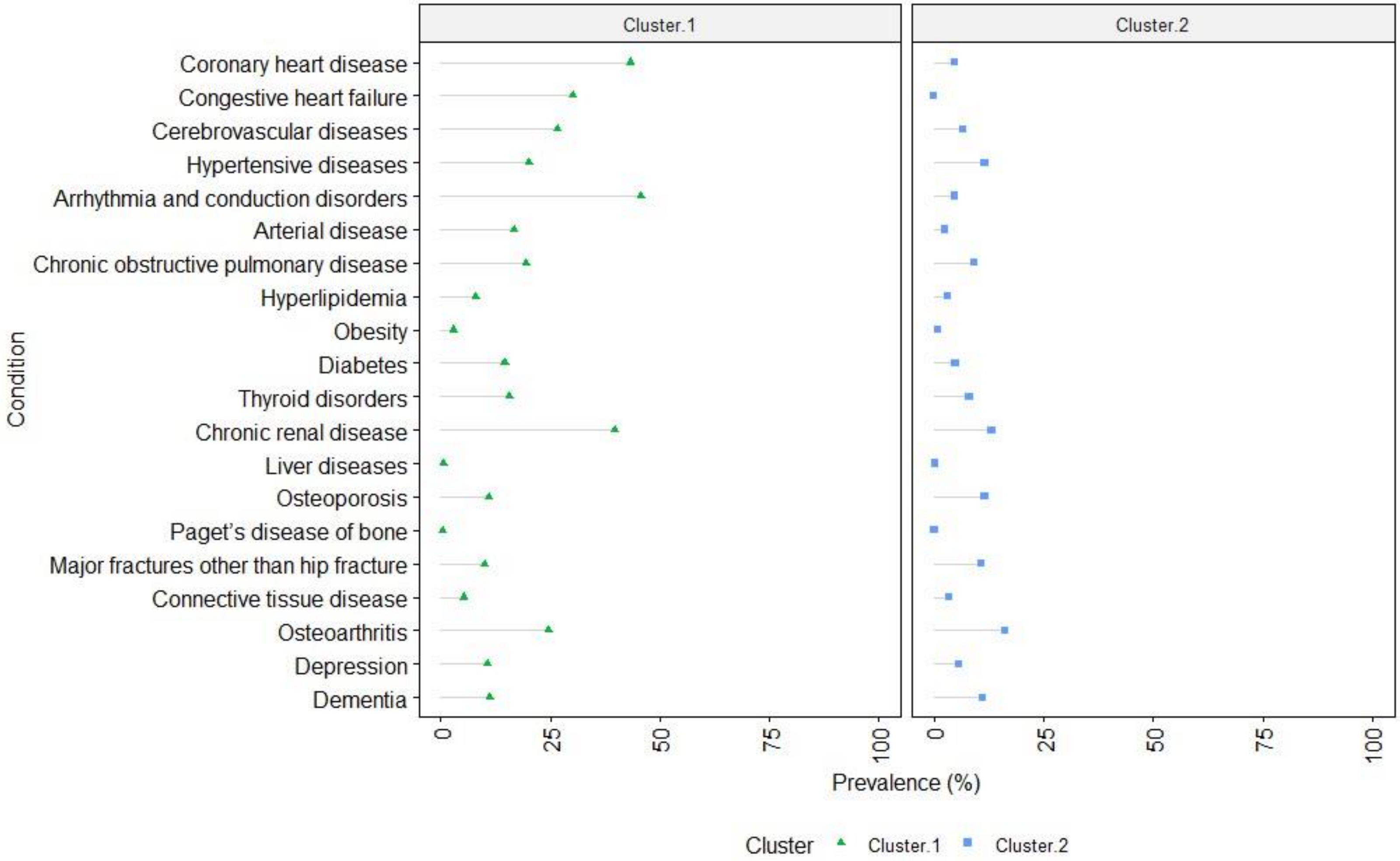

The Prevalence are indicated by the symbols (triangle for Cluster 1; Square for Cluster 2). n = 27,948 independent patients in UK THIN (n = 4,966 in Cluster 1; n = 22,982 in Cluster 2).

Supplementary Figure 4: Hazard ratios for MACE at multiple time points from competing risk regression.
(a) HK CDARS: Cluster 1 vs 3

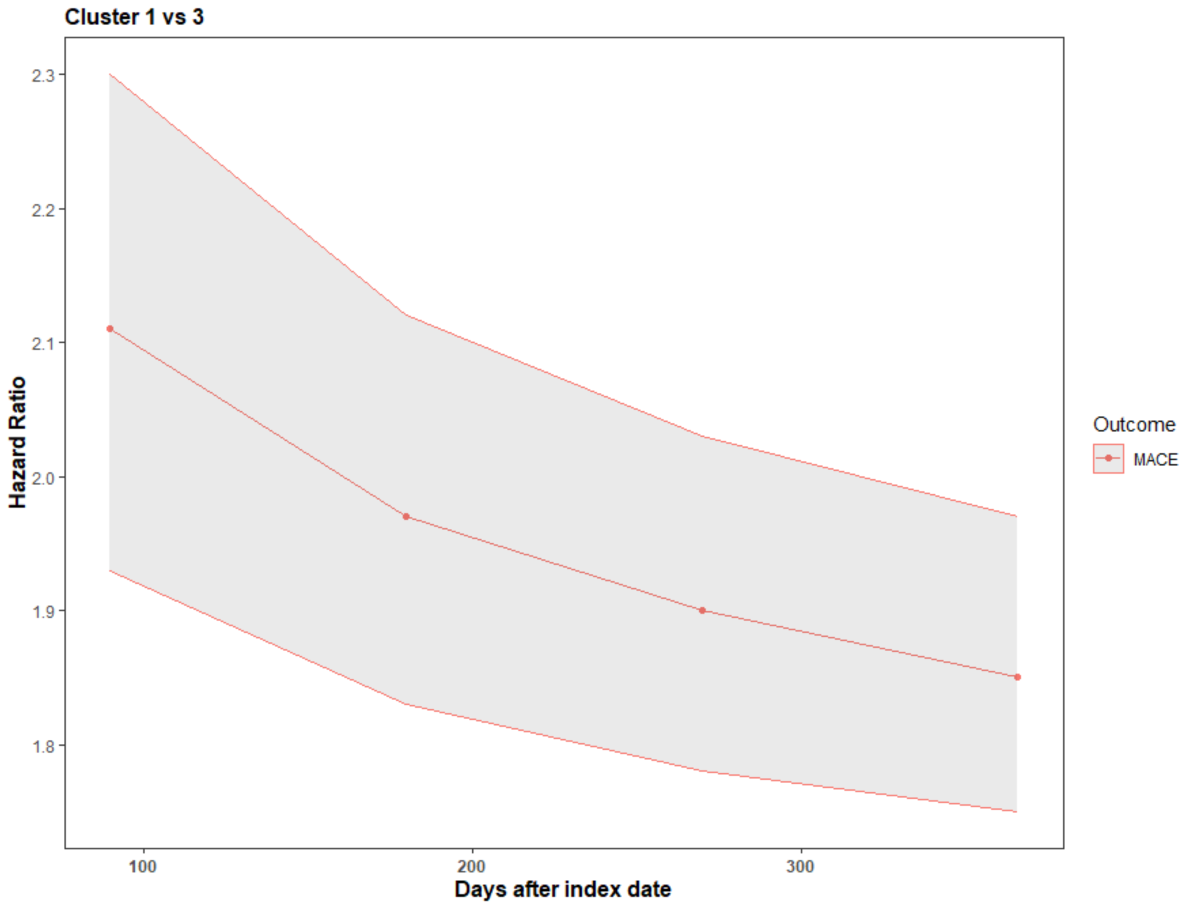

	Hazard Ratios from Competing Risk Regression (95% CI) p-value			
	90 days	180 days	270 days	1 year
MACE	2.11 (1.93 – 2.30) p < 0.001	1.97 (1.83 – 2.12) p < 0.001	1.90 (1.78 – 2.03) p < 0.001	1.85 (1.75 – 1.97) p < 0.001

Data are presented as Hazard Ratio (95% Confidence Interval) two-sided p-value derived from competing risk regression. All analyses are adjusted for age and sex. The Hazard Ratios are indicated by the central symbol, and the 95% CI are indicated by the error bar. Cluster 3 is the reference group. n = 16,762 independent patients in Cluster 1; n = 51,795 independent patients in Cluster 3. Note: MACE = Major Adverse Cardiovascular Events; CI = Confidence Interval.

(b) HK CDARS: Cluster 2 vs 3

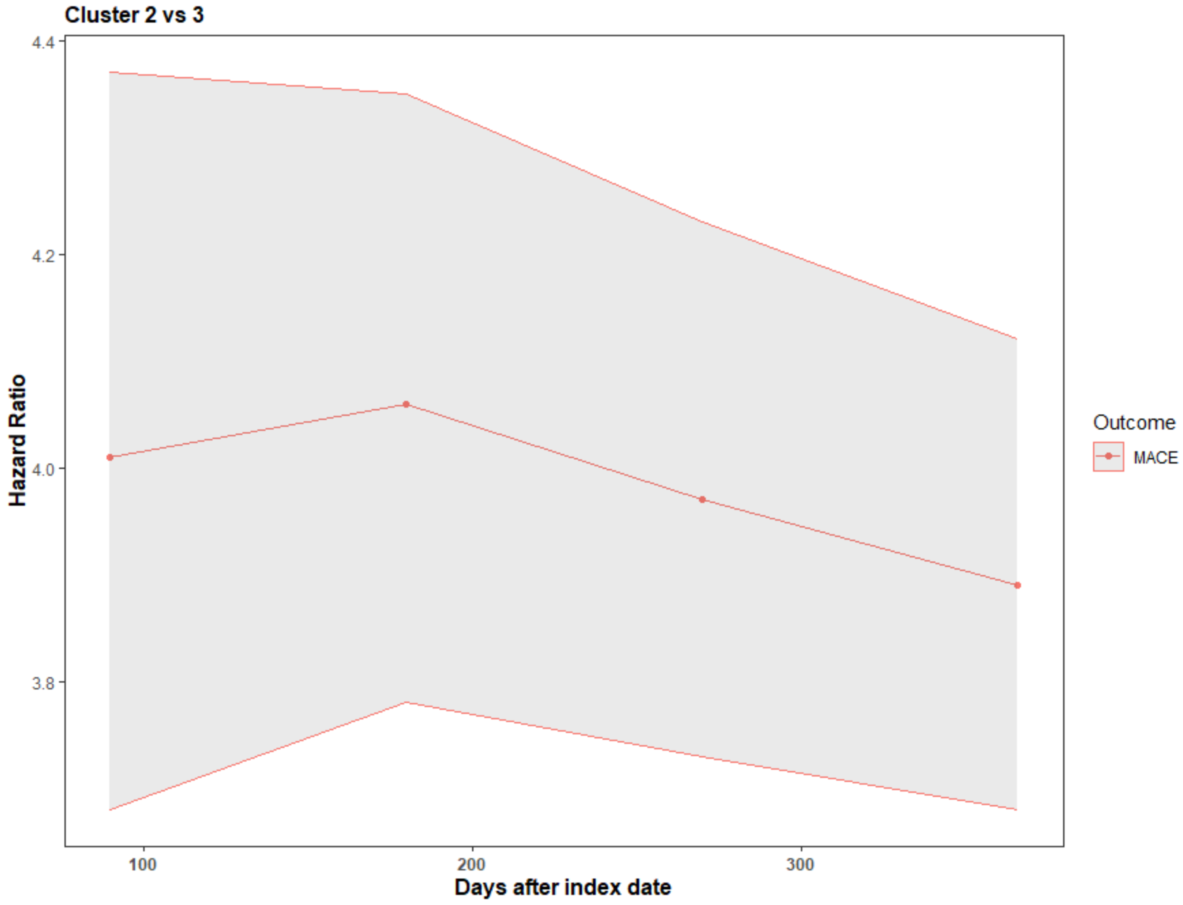

Hazard Ratios from Competing Risk Regression (95% CI) p-value				
	90 days	180 days	270 days	1 year
MACE	4.01 (3.68 – 4.37) p < 0.001	4.06 (3.78 – 4.35) p < 0.001	3.97 (3.73 – 4.23) p < 0.001	3.89 (3.68 – 4.12) p < 0.001

Data are presented as Hazard Ratio (95% Confidence Interval) two-sided p-value derived from competing risk regression. All analyses are adjusted for age and sex. The Hazard Ratios are indicated by the central symbol, and the 95% CI are indicated by the error bar. Cluster 3 is the reference group. n = 9,860 independent patients in Cluster 2; n = 51,795 independent patients in Cluster 3. Note: MACE = Major Adverse Cardiovascular Events; CI = Confidence Interval.

(c) UK THIN Cohort: Cluster 1 vs 2

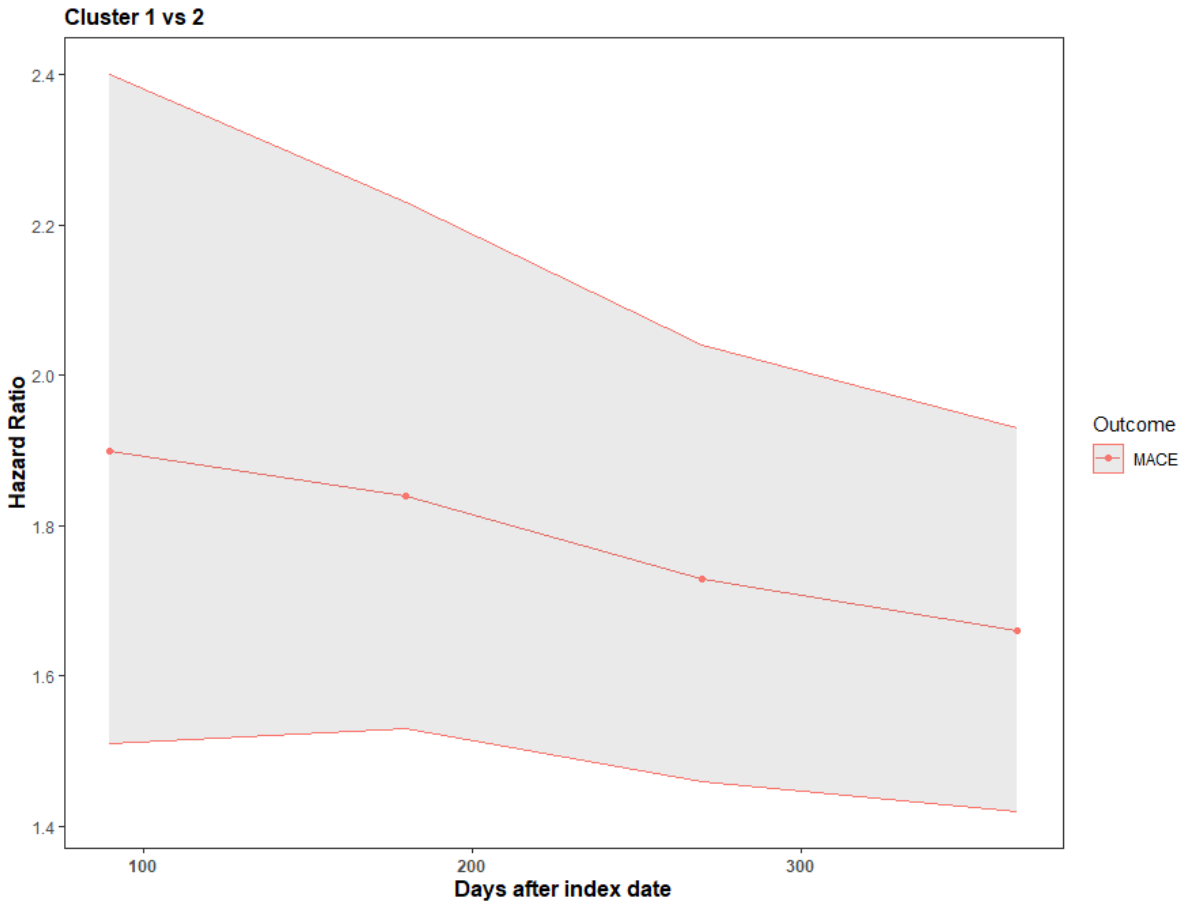

Hazard Ratios from Competing Risk Regression (95% CI) p-value				
	90 days	180 days	270 days	1 year
MACE	1.90 (1.51 – 2.40) p < 0.001	1.84 (1.53 – 2.23) p < 0.001	1.73 (1.46 – 2.04) p < 0.001	1.66 (1.42 to 1.93) p < 0.001

Data are presented as Hazard Ratio (95% Confidence Interval) two-sided p-value derived from competing risk regression. All analyses are adjusted for age and sex. The Hazard Ratios are indicated by the central symbol, and the 95% CI are indicated by the error bar. Cluster 2 is the reference group. n = 4,966 independent patients in Cluster 1; n = 22,982 independent patients in Cluster 2. Note: MACE = Major Adverse Cardiovascular Events; CI = Confidence Interval.

Supplementary Figure 5: Association between hip fracture and individual MACE outcomes through SCCS.
(a) HK CDARS

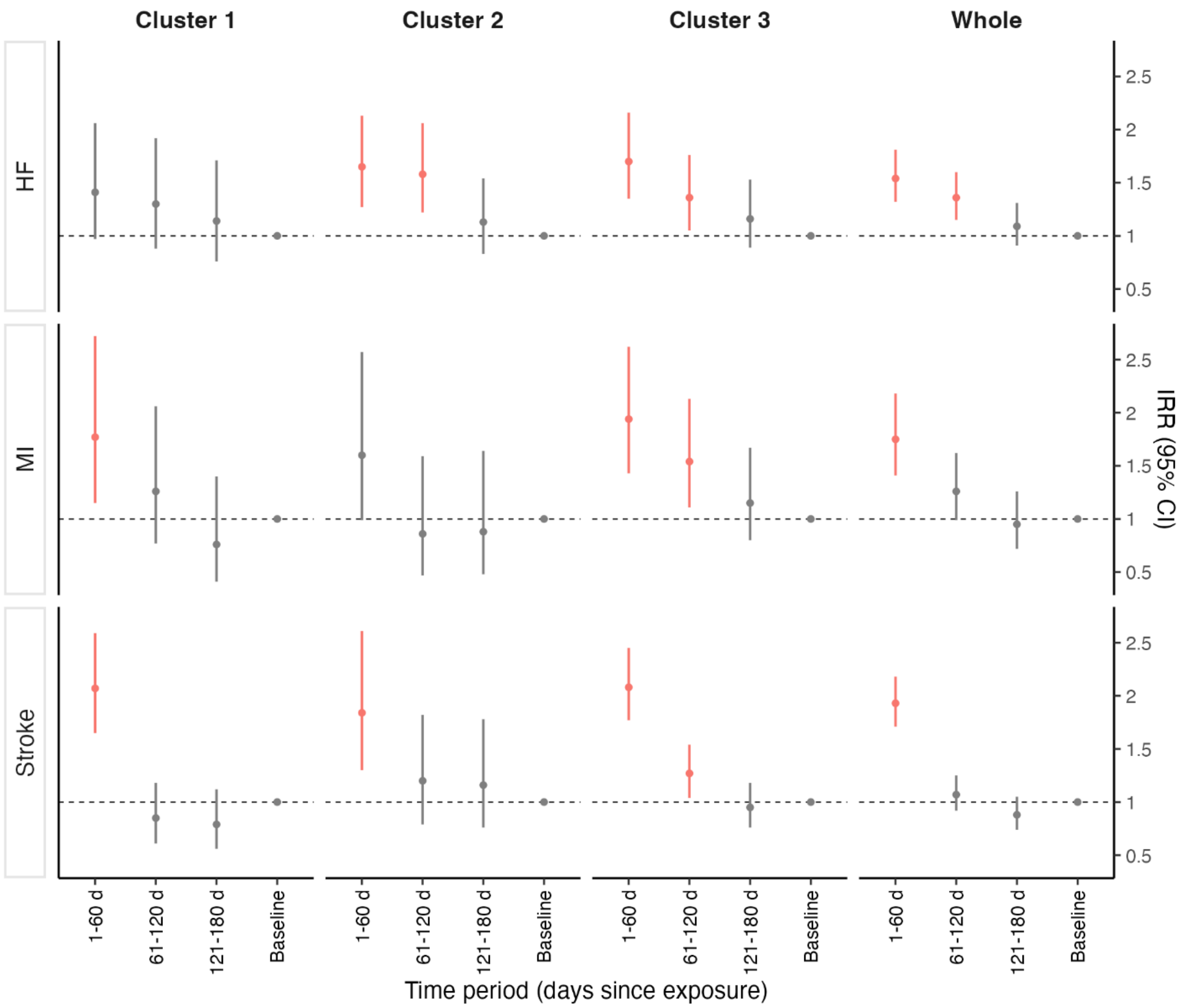

Notes: HF = heart failure hospitalisation; MI = myocardial infarction; IRR = Incidence Rate Ratios; CI = Confidence Interval.

(b) UK THIN

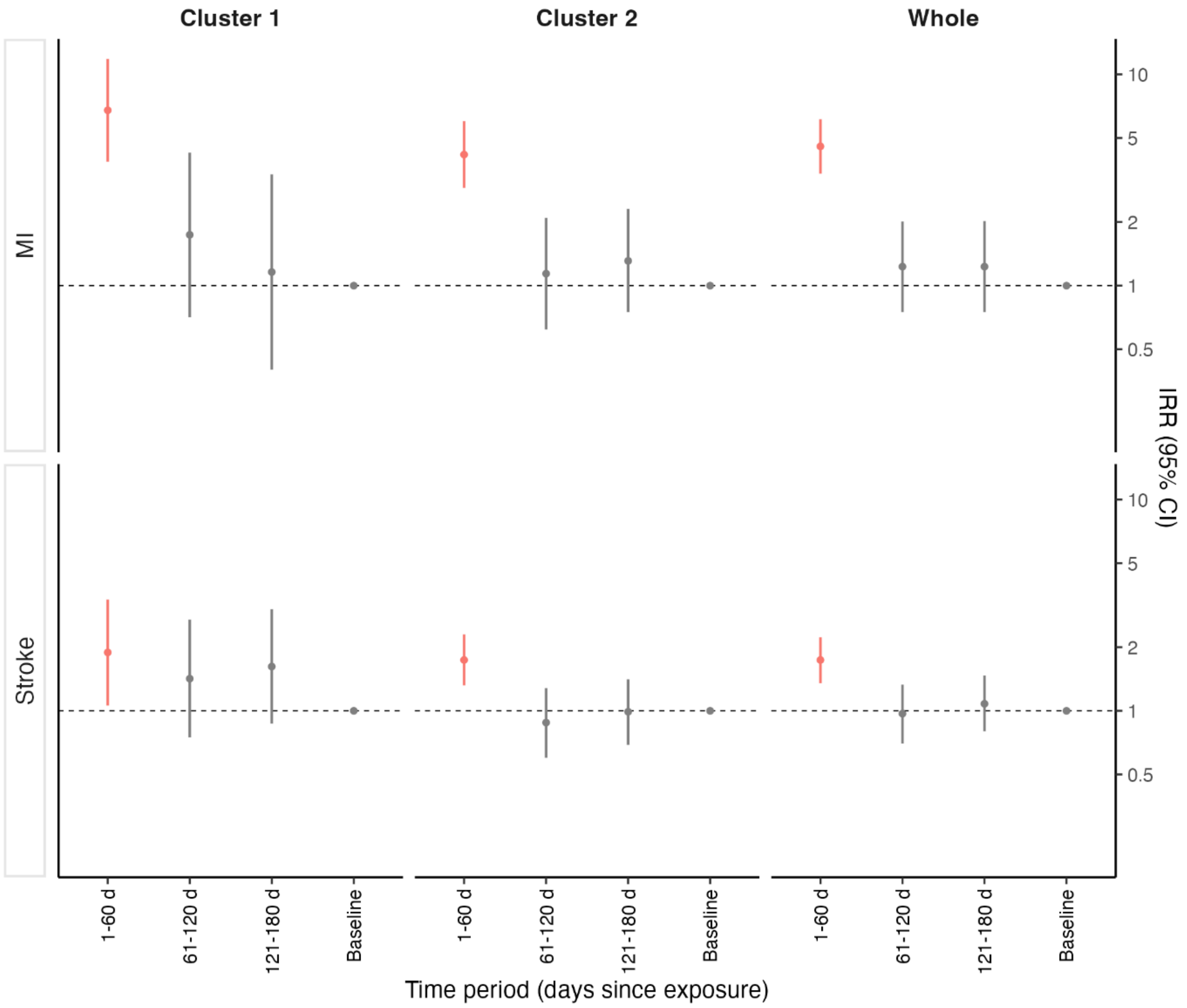

Notes: MI = myocardial infarction; IRR = Incidence Rate Ratios; CI = Confidence Interval.

Supplementary Figure 6. Incidence Rates for MACE by Cluster in the Hip Fracture Cohorts.

(a) HK CDARS

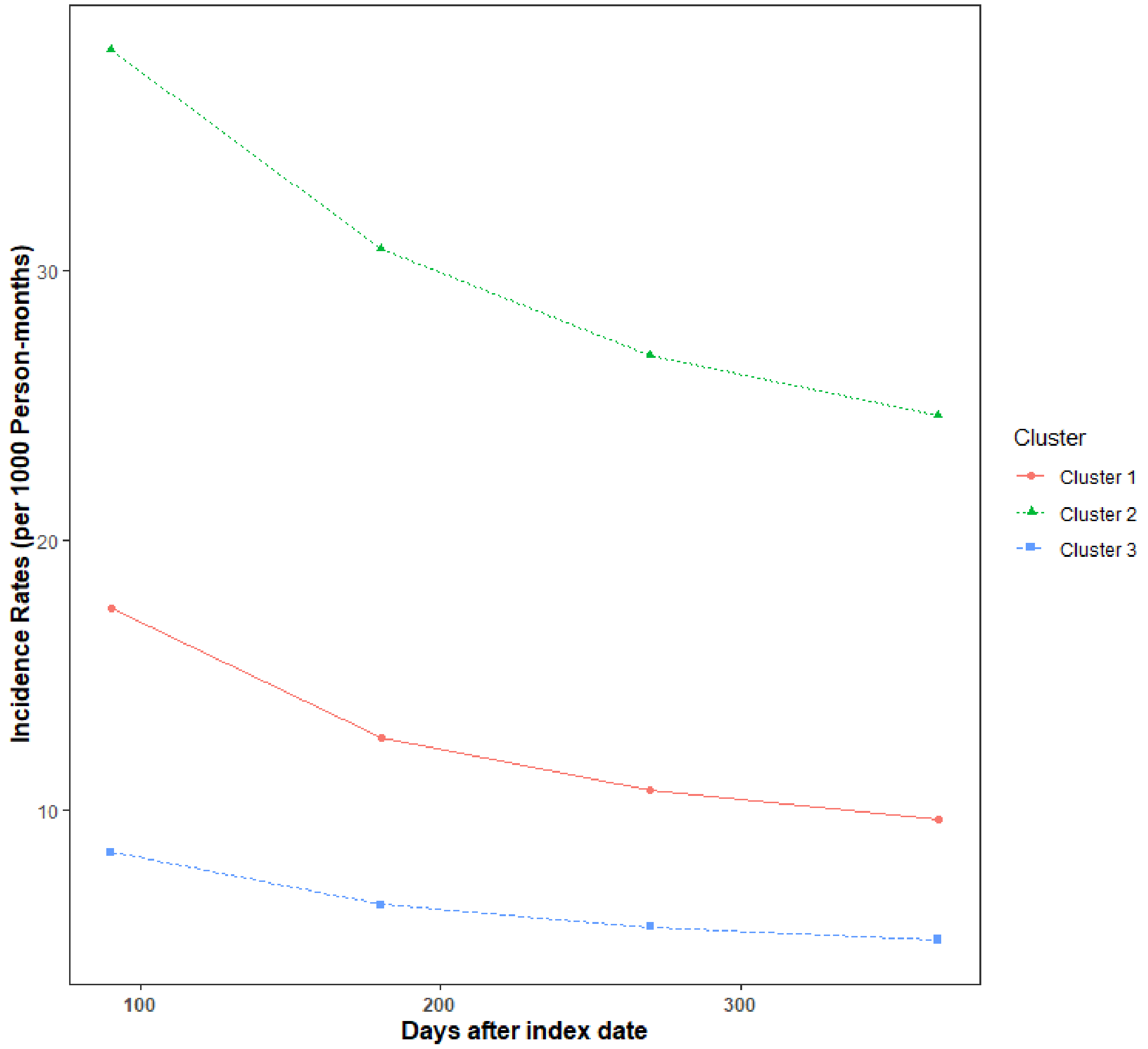

Incidence Rates for MACE (per 1000 Person-months)				
	90 days	180 days	270 days	1 year
Cluster 1	17.47	12.69	10.78	9.67
Cluster 2	38.16	30.76	26.85	24.61
Cluster 3	8.46	6.53	5.71	5.23

The Incidence Rates are indicated by the symbols (circle for Cluster 1; triangle for Cluster 2; square for Cluster 3). n = 78,417 independent patients in HK CDARS (n = 16,762 in Cluster 1; n = 9,860 in Cluster 2; n = 51,795 in Cluster 3). Note: MACE = Major Adverse Cardiovascular Events.

(b) UK THIN

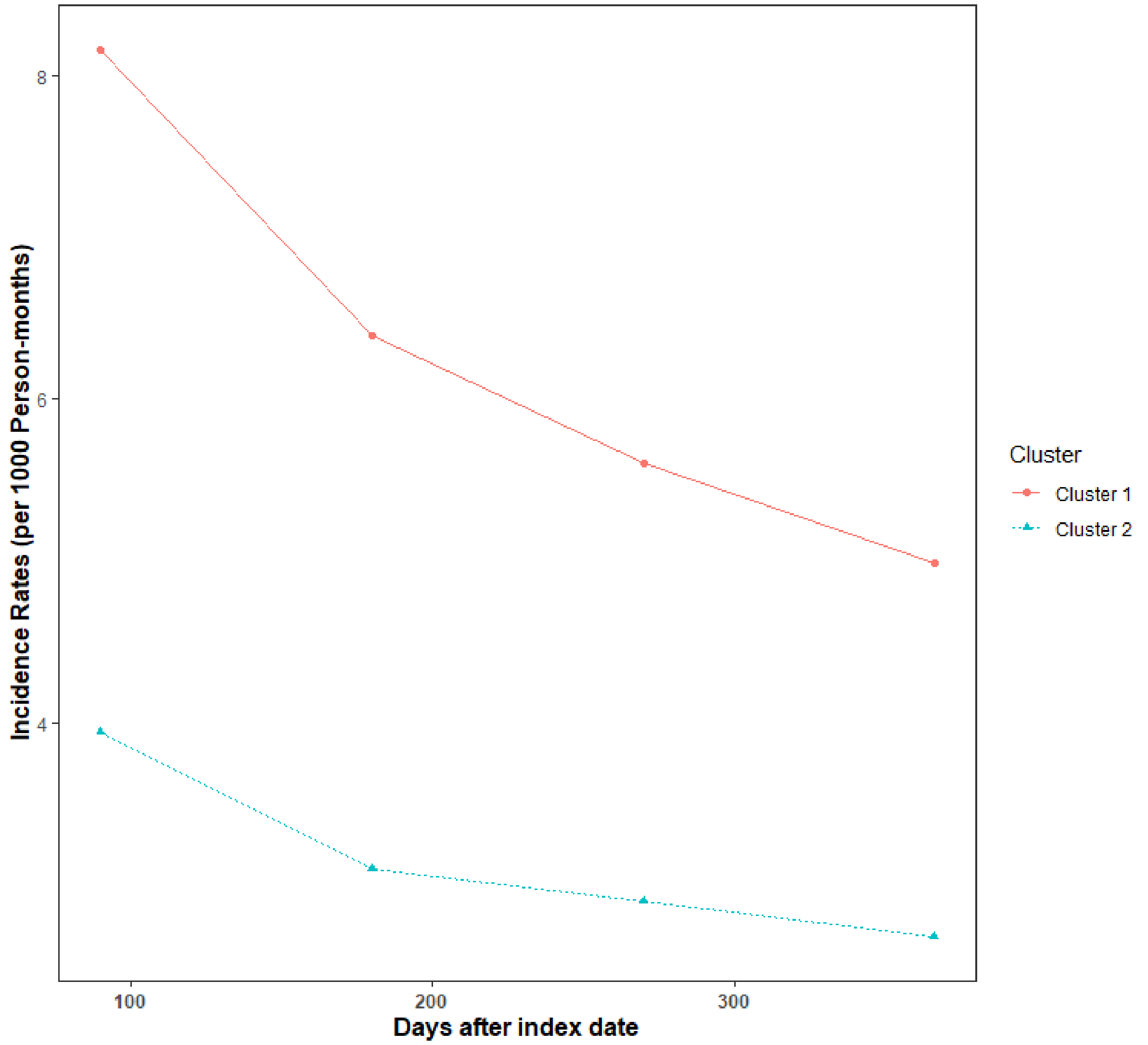

Incidence Rates for MACE (per 1000 Person-months)				
	90 days	180 days	270 days	1 year
Cluster 1	8.16	6.39	5.60	4.99
Cluster 2	3.94	3.10	2.90	2.68

The Incidence Rates are indicated by the symbols (circle for Cluster 1; triangle for Cluster 2). n = 27,948 independent patients in UK THIN (n = 4,966 in Cluster 1; n = 22,982 in Cluster 2). Note: MACE = Major Adverse Cardiovascular Events.

Supplementary Figure 7. Schematic plot of the SCCS design.

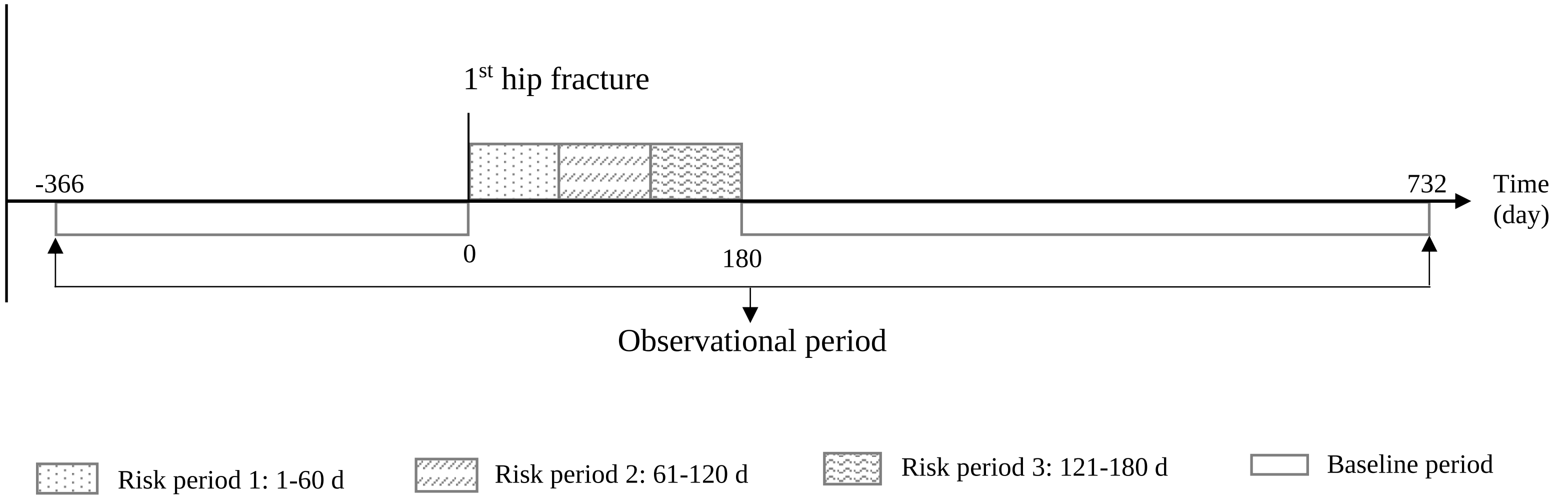

Supplement: Supplementary file 1 — Supplementary Information [file 41467_2024_48713_MOESM1_ESM.pdf]
