# Supplementary material for: Unveiling unique clinical phenotypes of hip fracture patients and the temporal association with cardiovascular events
Source: Nat Commun. 2024 May 22;15:4353. doi: 10.1038/s41467-024-48713-3 (PMC11111763; doi:10.1038/s41467-024-48713-3)
Supplement: Supplementary file 3 — Reporting summary [file 41467_2024_48713_MOESM3_ESM.pdf]

nature portfolio

Corresponding author(s): Prof. Ching-Lung Cheung

Last updated by author(s): Apr 28, 2024

Reporting Summary

Nature Portfolio wishes to improve the reproducibility of the work that we publish. This form provides structure for consistency and transparency in reporting. For further information on Nature Portfolio policies, see our Editorial Policies and the Editorial Policy Checklist.

Statistics

For all statistical analyses, confirm that the following items are present in the figure legend, table legend, main text, or Methods section.

n/a	Confirmed	
☐	☒	The exact sample size (*n*) for each experimental group/condition, given as a discrete number and unit of measurement
☐	☒	A statement on whether measurements were taken from distinct samples or whether the same sample was measured repeatedly
☐	☒	The statistical test(s) used AND whether they are one- or two-sided<br>*Only common tests should be described solely by name; describe more complex techniques in the Methods section.*
☐	☒	A description of all covariates tested
☐	☒	A description of any assumptions or corrections, such as tests of normality and adjustment for multiple comparisons
☐	☒	A full description of the statistical parameters including central tendency (e.g. means) or other basic estimates (e.g. regression coefficient) AND variation (e.g. standard deviation) or associated estimates of uncertainty (e.g. confidence intervals)
☐	☒	For null hypothesis testing, the test statistic (e.g. *F*, *t*, *r*) with confidence intervals, effect sizes, degrees of freedom and *P* value noted<br>*Give P values as exact values whenever suitable.*
☒	☐	For Bayesian analysis, information on the choice of priors and Markov chain Monte Carlo settings
☒	☐	For hierarchical and complex designs, identification of the appropriate level for tests and full reporting of outcomes
☐	☒	Estimates of effect sizes (e.g. Cohen's *d*, Pearson's *r*), indicating how they were calculated

Our web collection on statistics for biologists contains articles on many of the points above.

Software and code

Policy information about availability of computer code

Data collection	No software used for data collection.
Data analysis	This study did not generate any new algorithm/model. Statistical analyses were performed using R software (version 4.3.0; R Foundation for Statistical Computing, Vienna, Austria) through R packages poLCA (version 1.6.0.1) and the R script published by Lezhnina and Kismihok (2022) for the latent class analysis (LCA), the package “cmprsk” (version 2.2-11) was used to run the competing risk regression, and the package “SCCS” (version 1.6) was used for the SCCS analyses.

For manuscripts utilizing custom algorithms or software that are central to the research but not yet described in published literature, software must be made available to editors and reviewers. We strongly encourage code deposition in a community repository (e.g. GitHub). See the Nature Portfolio guidelines for submitting code & software for further information.

Data

Policy information about availability of data

All manuscripts must include a data availability statement. This statement should provide the following information, where applicable:

- Accession codes, unique identifiers, or web links for publicly available datasets
- A description of any restrictions on data availability
- For clinical datasets or third party data, please ensure that the statement adheres to our policy

The data used in this study cannot be shared with the public due to third-party use restrictions and patient confidentiality concerns. The HK CDARS EHR database is directly under the control of the Hong Kong Hospital Authority. Local academic institutions, government departments, or non-governmental organisations can apply

April 2023

for access to CDARS data through Hong Kong Hospital Authority Data Sharing Portal (https://www3.ha.org.hk/data). The detailed application procedure can be found at https://www3.ha.org.hk/data/Provision/ApplicationProcedure. The UK THIN, a Cegedim EHR Database, is licensed by IQVIA. It is available for researchers from academic, public health, research establishment, charitable, commercial and regulatory bodies through purchase. Information on IQVIA Medical Research Data (IMRD) which incorporated the UK THIN data, can be found at https://www.iqvia.com/locations/united-kingdom/information-for-members-of-the-public/medical-research-data. Applications to access the UK THIN data can be made via https://www.the-health-improvement-network.com/.

Research involving human participants, their data, or biological material

Policy information about studies with human participants or human data. See also policy information about sex, gender (identity/presentation), and sexual orientation and race, ethnicity and racism.

Reporting on sex and gender	Both sexes were considered in the study design with the proportion of males and females reported. The sex was determined based on the information health professionals recorded in the electronic health record databases. 31778 males and 74587 females involved in the cohorts. Sex was included as a clustering variable in the latent class analysis, meaning that sex was considered when using our LCA model to assign a patient to the optimal patient cluster. Furthermore, a survival analysis stratified by sex was conducted to evaluate potential sex-specific risk of health outcomes between the patient clusters identified.
Reporting on race, ethnicity, or other socially relevant groupings	No social constructed variables involved in the study. The races are mostly Asians and White. Sex and age were included as clustering variables in LCA, and were considered as confounding variables adjusted in the cox proportional and competing risk models. The SCCS inherently controlled for the time-invariant covariates at the individual level.
Population characteristics	The summary of the populations characteristics (e.g., age, sex, and baseline diagnoses) were provided in Supplementary Table 1.
Recruitment	The cohorts were retrieved directly from the electronic health records (EHR). No self-selection bias was involved. However, severe conditions that were more likely to be associated with hospitalisation might be expected to be captured more comprehensively and timely in the hospital-based CDARS, when compared to the primary care-based THIN. Underreporting of diagnoses in EHR databases might in general potentially bias risk estimation, but was more likely to be an underestimation.
Ethics oversight	Ethical approval for this study was granted by the Institutional Review Board (IRB) of The University of Hong Kong/HA HKW, Hong Kong Special Administrative Region and the Scientific Review Committee (SRC) in the UK. The informed consent was exempted as all patients were non-identifiable in the population-based study.

Note that full information on the approval of the study protocol must also be provided in the manuscript.

Field-specific reporting

Please select the one below that is the best fit for your research. If you are not sure, read the appropriate sections before making your selection.

☒ Life sciences ☐ Behavioural & social sciences ☐ Ecological, evolutionary & environmental sciences

For a reference copy of the document with all sections, see nature.com/documents/nr-reporting-summary-flat.pdf

Life sciences study design

All studies must disclose on these points even when the disclosure is negative.

Sample size	The sample size was determined by identifying the number of patients aged 65 years or older with newly diagnosed hip fractures recorded in the HK CDARS and UK THIN electronic health record databases. This resulted in a total sample size of 106,365 (78,417 in HK CDARS and 27,948 in UK THIN). CDARS covers >80% of hospital admissions in HK, and around 98% of hip fracture cases in HK were admitted to hospitals under the Hospital Authority. UK THIN covers about 6% of the population with more than 700 general practitioner practices in the UK. Both population-based EHR databases have been used to conduct population-wide observational studies associated with hip fracture.
Data exclusions	No data were excluded from the analyses.
Replication	The analyses were independently performed twice: once on the HK CDARS cohort and once on the UK THIN cohort. The consistent results suggest the reproducibility and generalisability of the findings.
Randomization	NA. This is a retrospective population-based study. No allocation of treatment was involved.
Blinding	NA. No allocation was involved and all individuals in the databases were deidentified.

Reporting for specific materials, systems and methods

We require information from authors about some types of materials, experimental systems and methods used in many studies. Here, indicate whether each material, system or method listed is relevant to your study. If you are not sure if a list item applies to your research, read the appropriate section before selecting a response.

Materials & experimental systems

n/a	Involved in the study
☒	☐ Antibodies
☒	☐ Eukaryotic cell lines
☒	☐ Palaeontology and archaeology
☒	☐ Animals and other organisms
☒	☐ Clinical data
☒	☐ Dual use research of concern
☒	☐ Plants

Methods

n/a	Involved in the study
☒	☐ ChIP-seq
☒	☐ Flow cytometry
☒	☐ MRI-based neuroimaging

Plants

Seed stocks	NA
Novel plant genotypes	NA
Authentication	NA
